# Tactical games model in physical education: A systematic review

**DOI:** 10.1371/journal.pone.0311321

**Published:** 2024-11-08

**Authors:** Jiaxu Wang, Chee Chen Soon, Shamsulariffin Samsudin, Chen Wang, Zhendong Gao, Qian Xie

**Affiliations:** 1 Faculty of Educational Studies, Department of Sports Studies, Universiti Putra Malaysia, Selangor, Serdang, Malaysia; 2 School of Physical Education, Shaanxi Normal University, Xi’an, China; University of Study of Bari Aldo Moro, ITALY

## Abstract

Since the Tactical Games Model (TGM) was adopted and popularised by Oslin, Griffin, and Mitchell, research interest in this model has surged, increasing its application in teaching and research. However, much-existing research is fragmented and lacks a comprehensive review. This systematic review aims to fill that gap by thoroughly analysing the literature on TGM within the context of physical education, highlighting current trends and developments. We systematically searched four online databases, identifying 38 relevant articles for inclusion, which were then evaluated across eight domains following PRISMA guidelines. The analysis indicates a steady increase in TGM-focused research within physical education, particularly at the K-12 level. However, there is a notable absence of studies addressing higher education, as well as teachers and coaches. The review also found that the majority of TGM research is concentrated on team sports and invasion games, with limited exploration of other sports. The research objectives often centred on extrinsic factors, such as game performance, skill level, and physical activity (PA) level, as well as intrinsic factors, intrinsic motivation, stress, and enjoyment. The TGM has shown varying levels of success in improving these factors. Despite the recognised benefits of TGM in physical education, its impact on cognitive aspects and decision-making remains underexplored. This systematic review may serve as a valuable resource for educators and researchers, supporting the broader application of TGM across different areas of physical education and potentially leading to enhanced student outcomes.

## 1 Introduction

In recent decades, physical education has received increasing attention in the educational community [1], and many scholars have conducted a great deal of research on physical education pedagogy [2–5]. Most of these teachers and researchers have focused on technology and skill-centred teaching methods, which have been the most widely used in physical education, usually with a focus on technique, where teachers teach fixed techniques and skills and then require students to perform pre-defined exercises for repetition in order to achieve perfect replication of the techniques and skills [6–10]. Moreover, it will isolate the environment for sports activities such as competitions from practice, where students must learn a certain level of technique and skill first [11,12]. However, the demand for mastery of skills in sports is not only physical or technical skills [13–15]. At the same time, criticisms of the technique and skills-based teaching approach suggest that this approach limits students’ initiative in their learning, makes them too dependent on the teacher, and also lacks the transition between practice and competition, allowing students to learn to develop a single, rigid technique, detached from real competition situations, unable to apply skills to real competition flexibly and reasonably solve problems that arise in competition [16–18]. To address these shortcomings in a targeted manner, teachers and researchers have been developing new concepts of teaching models and searching for and developing other effective teaching methods [5,19–21]. The concept of student-centred teaching and learning focused on play was first introduced in the 1970s, Following Bunker and Thorpe’s introduction of the concept of “Teaching Games for Understanding” (TGFU) in 1982 [22], many teaching and training methods based on games have been developed. Many studies have evaluated the effectiveness of these teaching models, and these studies have confirmed that teaching physical education through games can positively impact teaching effectiveness. These studies have also confirmed the positive impact that was teaching through games can have on teaching and learning [7,12,23,24], but the question of which approach to teaching games is most effective remains a controversial one worldwide [25].

The Tactical Games Model (TGM), developed as a teaching model based on the TGFU, is an increasingly mainstream way of teaching lessons through games in physical education [26]. The theoretical content of the TGM is based on an extension and simplification of the TGFU theory, focusing on tactical problem-solving by students [27,28], in which the TGM simplifies the six stages of the TGFU teaching model into three stages, namely:

1. Start the course with a modified form of exaggerated play.
2. Cultivate students’ tactical awareness by asking questions to make them aware of the skills and techniques required while allowing them to make their own decisions to solve tactical problems as they arise.
3. Develop skills, practice and play again [29].

At the same time, there has been increased research on TGM in recent years, with researchers replacing traditional teaching models by using TGM for instruction and achieving specific learning outcomes [23]. Firstly, students can exercise and develop a sense of play by participating in a modified tour in the TGM [23,30]. Secondly, this exciting teaching method can also enhance the motivation of primary school students towards learning [31,32]. Although there has been a progressive increase in research on TGM, these studies are more fragmented and lack a systematic review of TGM in physical education. Therefore, the primary purpose of this study was to review the literature on research on the Tactical Games Model in the field of physical education and to systematically collate and analyse these studies for the benefit of teachers and researchers conducting TGM research and implementation in the future.

## 2 The review

### 2.1 Aim of review

This research reviews studies concerning the Tactical Games Model in physical education. According to this objective, the following questions were addressed in this review:

1. What are the current research trends in the tactical games model in physical education?
2. Who were the study participants?
3. What are the main objectives of TGM research within physical education?
4. What domains of the Tactical Games Model are being researched?
5. What assessment instruments have been used to study TGM in physical education?
6. What are the results of the study?

### 2.2 Review design

This systematic review was conducted following the Guidelines for the Preferred Reporting Item for the Protocol for Systematic Reviews and Meta-Analysis (PRISMA) [33]and the PICO Search Strategy [34]. This review summarises previous research findings and provides a theoretical basis and research framework for studying TGM in physical education.

### 2.3 Review strategy

This search conducted a systematic literature search in four electronic databases: Web of Science (WOS), Scopus, Taylor & Francis and ERIC, using "Tactical Games Model", "Tactical Games Approach", and "TGM" as the primary search terms and the search operator "or". The specific search operator was All = (("tactical games model" OR "TGM" OR "tactical games approach") AND ("physical education" OR sport*)). The Web of Science was chosen because it includes over 1 billion cited references in its data and provides a citation service of over 65 million citations per year. It is also the largest accessible citation database. The Scopus was chosen because it includes over 5,000 publishers worldwide in science, technology, medicine, and social sciences has a good reputation worldwide. Taylor & Francis was chosen because it is not only the largest global academic publisher in the humanities and social sciences with many journals but also contains over 48,000,000 articles covering a wide range of research areas. The ERIC was chosen because it is the largest online database in the field of education, with an authoritative educational index and a large number of educational literature resources.

### 2.4 Quality appraisal

To reduce the risk of review bias, two independent researchers used the PEDro scale, built on the Physiotherapy Evidence Database, to assess the quality of studies included in the review [35]. The PEDro scale has 11 items to evaluate the quality of the study, and items that meet the criteria will be awarded 1 point. Except for the first evaluation item in the scale, the research will be evaluated on a scale of 0–10—the higher scores indicating a better quality study. The quality of the included reviews in this study was independently reviewed by two authors. In case of disagreement with the evaluation results, they were still jointly evaluated and resolved with the third author. Table 1 shows the quality evaluation results of the included articles.

**Table 1:** Quality evaluation according to the PEDro scale.

Quality Evaluation Form												
Author/Published Year	1	2	3	4	5	6	7	8	9	10	11	Score
MacPhail, Kirk [36]	Y	Y	Y	Y	Y	N	N	N	Y	Y	Y	8
Oh, Bullard [37]	N	Y	Y	N	Y	N	Y	N	Y	Y	Y	7
Thomas, Morgan [38]	Y	Y	Y	Y	Y	N	Y	Y	Y	Y	N	8
Harvey, Smith [32]	Y	Y	Y	N	Y	N	Y	Y	Y	Y	Y	8
Smith, Harvey [39]	Y	Y	Y	Y	Y	N	Y	Y	Y	Y	Y	9
Harvey and Pill [40]	Y	Y	Y	N	Y	N	Y	N	Y	N	Y	6
Harvey, Smith [41]	Y	Y	Y	Y	N	N	Y	N	Y	Y	Y	7
Harvey and Atkinson [42]	Y	N	N	Y	Y	N	Y	Y	Y	Y	N	6
Harvey and García-López [43]	Y	Y	Y	Y	Y	N	Y	Y	Y	Y	Y	9
Harvey, Gil-Arias [31]	Y	Y	Y	Y	Y	N	Y	Y	Y	Y	N	9
Hodges, Wicke [30]	Y	Y	Y	Y	Y	N	Y	Y	Y	Y	Y	9
Williams and Hannon [44]	Y	Y	Y	Y	Y	N	Y	Y	Y	Y	Y	9
Alagül and Gürsel [45]	Y	N	N	Y	Y	N	N	N	Y	Y	Y	5
Garcia-Ceberino, Feu [46]	Y	Y	Y	Y	Y	N	Y	Y	Y	Y	Y	9
Gouveia, Gouveia [47]	Y	Y	Y	Y	Y	N	Y	Y	Y	Y	Y	9
Güneş and Yılmaz [48]	Y	Y	Y	Y	Y	N	Y	Y	Y	Y	Y	9
Rodríguez-Negro, Falese [49]	Y	Y	Y	Y	Y	N	Y	Y	Y	Y	Y	9
Coppola, Pignato [50]	Y	Y	Y	Y	Y	N	Y	Y	Y	Y	Y	9
García-Ceberino, Antúnez [51]	Y	Y	Y	Y	Y	N	Y	Y	Y	Y	Y	9
García-Ceberino, Gamero [52]	Y	Y	Y	Y	Y	N	Y	Y	Y	Y	Y	9
García-Ceberino, Gamero [53]	Y	Y	Y	Y	Y	N	Y	Y	Y	Y	Y	9
Gonzalez-Espinosa, Antunez [54]	Y	Y	Y	Y	Y	N	Y	Y	Y	Y	Y	9
González-Espinosa, García-Rubio [55]	Y	Y	Y	Y	Y	N	Y	Y	Y	Y	Y	9
Rodríguez-Negro and Yanci [56]	Y	Y	Y	Y	Y	N	Y	N	Y	Y	Y	8
Sgrò, Barca [57]	Y	Y	Y	Y	Y	N	Y	N	Y	Y	Y	8
SgrÒ, Coppola [58]	Y	Y	Y	Y	Y	N	Y	Y	Y	Y	Y	9
Gamero, Garcia-Ceberino [59]	Y	Y	Y	Y	Y	N	Y	Y	Y	Y	Y	9
Gamero, García-Ceberino [60]	Y	Y	Y	Y	Y	N	Y	Y	Y	Y	Y	9
Gonzalez-Espinosa, Garcia-Rubio [61]	Y	Y	Y	Y	Y	N	Y	Y	Y	Y	Y	9
Schembri, Coppola [62]	Y	Y	Y	Y	Y	N	Y	Y	Y	Y	Y	9
Sgrò, Coppola [63]	Y	Y	Y	Y	Y	N	Y	Y	Y	Y	Y	9
Feu, Garcia-Rubio [64]	Y	N	N	Y	N	Y	Y	N	Y	Y	Y	6
Garcia-Ceberino, Feu [65]	Y	Y	Y	Y	Y	N	Y	N	Y	Y	Y	8
Setiawan and Juliantine [66]	Y	Y	N	Y	Y	N	Y	Y	Y	Y	Y	8
Rodríguez-Negro and Yanci [67]	Y	Y	Y	Y	Y	N	Y	N	Y	Y	Y	8
	Y	Y	Y	Y	Y	N	Y	Y	Y	Y	Y	9
Hartati, Ursa [68]	Y	Y	Y	Y	Y	N	Y	Y	Y	Y	Y	9
Sucipto, Hambali [69]	Y	Y	Y	Y	Y	N	Y	Y	Y	Y	Y	9

Note: Y: Yes; N: No; 1: Eligibility criteria were determined; 2: The samples were randomly assigned; 3: The sample was concealed; 4: The groups were similar at baseline in terms of the most important prognostic indicators; 5: All samples were blinded; 6: Therapists were blinding through the intervention; 7: Evaluators were blinding for all measurements of at least one key outcome; 8: More than 85% of subjects initially assigned to each group obtained at least one key outcome measure; 9: All participants who received the assigned treatment or control condition had outcome measures, of which data for at least one key outcome was analysed by “intentional treatment”; 10: Report the results of statistical comparisons between groups for at least one key outcome; 11: The study provided point measurements and variability measures for at least one key outcome; total score: Except for the first item which is not scored, each yes item is awarded one mark towards the total score.

### 2.5 Inclusion criteria

Manuscripts that meet the following criteria are considered eligible for inclusion: (1) Articles written and published in English; (2) TGM-related research in the field of physical education; (3) Explicitly, the study of the Tactical Games Model; (4) The research is an independent study, rather than a systematic review or literature analysis of TGM. In order to reduce errors, authors reviewed each study separately. Finally, they aggregated and screened eligible literature, and if different literature existed, a third author reviewed it and consulted with both authors on whether to include it.

### 2.6 Exclusion criteria

Manuscripts meeting the following criteria were excluded: (1) Studies where the author of the article is unknown; (2) Articles with duplicate content, such as research studies where the same content is published in journals as papers and articles, collect incomplete data; (3) Articles for which full text is not available; (4) Articles not written in English.

### 2.7 Study selection and outcomes

**Fig 1:**
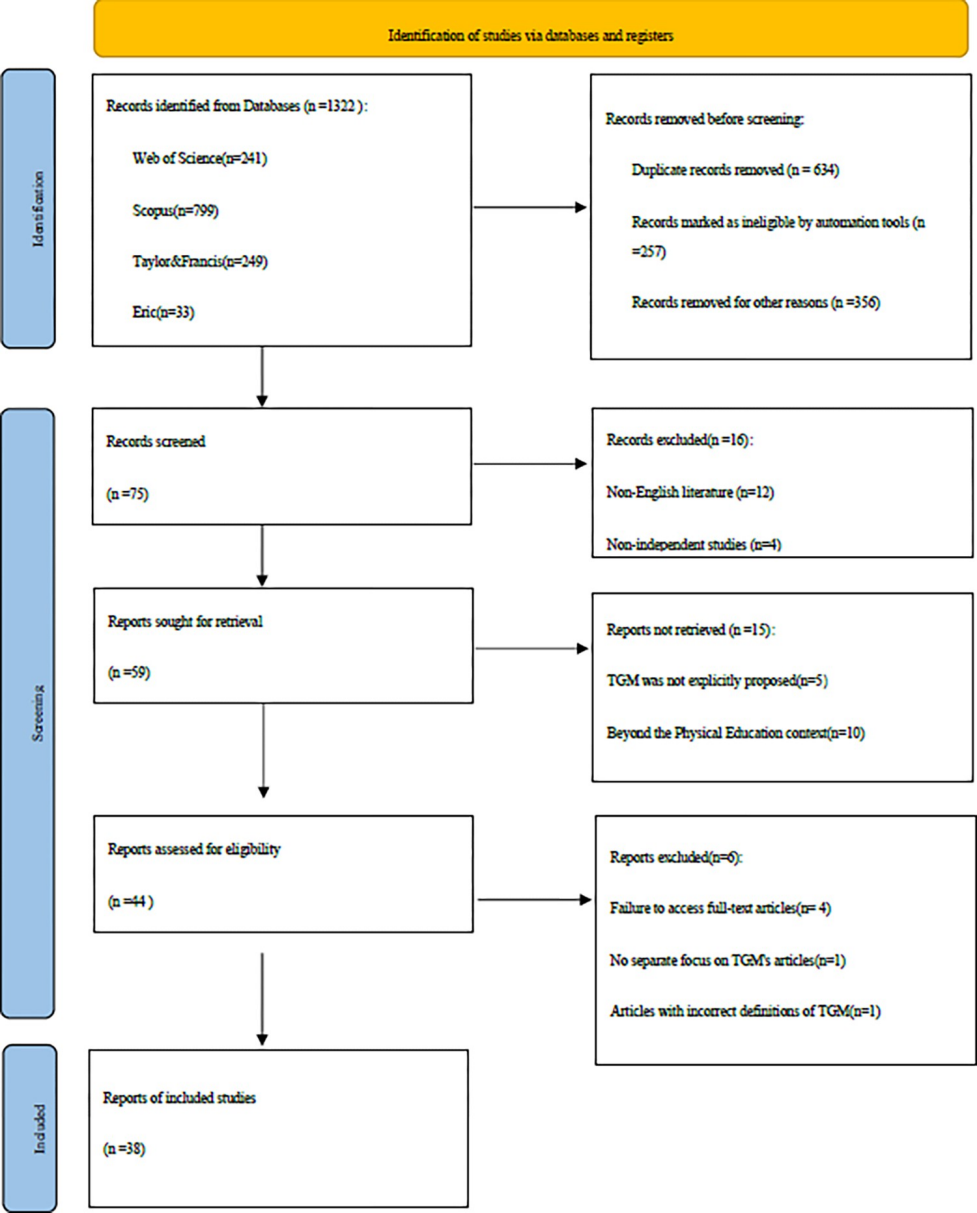
Identification of studies via databases.

In this review, we set the search time to January 2024. All articles were extracted from the database and analyzed by Endnote version 20 [70], and the search results were 1332 publications (Fig 1). The search results of each database are:Web of Science: 241; Scopus: 799; Taylor & Francis: 249; ERIC: 33. Two reviewers screened the existing literature according to the inclusion criteria and found 59 manuscripts that met the criteria. Then 44 publications were screened according to the exclusion criteria. For these 44 publications that met the criteria, two reviewers read and analyzed them in full and excluded six publications for the following reasons: Four articles could not be successfully accessed in full for review. One article did not conduct a separate study of TGM that was mixed with other teaching methods. In addition, one article incorrectly defined TGM and was not a study of the Tactical Games Model. Finally, a total of 38 articles were included for review (Table 2).

**Table 2:** Literature search results.

Database	Number of articles	Search terms
Web of Science	241	Tactical Games Model, TGM Tactical Games Approach
Scopus	799	
Taylor & Francis	249	
Eric	33	
Total	1322	
Preliminary review to removed	1263	
Studies selected after detailed review	59	
Studies eventually included in the review	38	

### 2.8 Data abstraction

Three reviewers conducted a thorough and systematic review of the 38 articles that were ultimately included in the review and produced the following data summary table. The following constructs were used: (1) authors of the articles and year of publication; (2) study context (country of study, intervention); (3) study participants (number, level of education); (4) type of study; (5) domain of application of the study; (6) objectives of the study; (7) assessment instruments; and (8) results of the study (Table 3).

**Table 3:** Summary of research data.

Author/Year	Context (country, intervention)		Participants (number, educational level)	Type of study	Application Domains	Research objectives	Assessment instruments	Results
MacPhail, Kirk [36]	GB; TGA	29; Primary school students (5^th^ grade)		Mix: QT: A non-intervention pedagogical experiment investigating contextual learning in a modified play unit QL: Naturalistic observations and descriptions of play; naturalistic and semi-structured individual and group interviews with learners and teachers	Invasion game unit	Describe and analyse how children learn to throw and catch in game situations involving attackers and defenders; make some observations about the impact of using the TGM methodology to construct learning experiments and assessments of physical education teaching	Semi-structured interviews with teachers and students (6 males and 6 females) in selected 3-to-3 groups GPAI to measure the game performance of 6 target learners	Game performance cannot improve significantly in a short period, but TGM positively impacts students’ throwing and catching
Oh, Bullard [37]	US; TGA	None; Middle school student		QL: Descriptive study	Speedminton	Introducing the non-traditional sport of badminton using a tactical games approach	Monitor and Assess	Benefits of teaching speed badminton using the tactical games approach: (1) Increased opportunities for physical activity enhanced physical fitness; (2) Improve students’ ability to use the tactical and strategic concepts of the game; (3) Provides opportunities to enjoy new games (4) Learning new ways to exercise throughout life
Thomas, Morgan [38]	GB; TGA	15–22; Primary school students (5^th^ and 6^th^ grades)		QL:Semi-structured group and individual interviews; reflective diary; Two semi-structured groups and six individual interviews were conducted with six players	Rugby/ Training: extracurricular activities	Reflect and analyse the implementation of TGA in coaching and the players’ learning experiences	Interview	TGA stimulated player interest, engagement and motivation in trainingHowever, players preferred to develop technical skills first, and TGA was inconsistent in developing players’ understanding of the game and decision-making competence, possibly due to the short duration of the intervention
Harvey, Smith [32]	GB; TGM/DIM	32; Middle school students (7^th^ grade)		QT: Quasi-experimental pre-post-test design	Hockey	Investigating students’ MVPA and VPA levels in DIM and TGM-focused PE units	RT3 Triaxial Accelerometry	Students in the TGM group had better levels of MVPA and VPA
Smith, Harvey [39]	GB; TGM/DIM	72; Middle school students (7^th^ grade)		QT: Pre-post-test quasi-experimental design	Netball/ Football/ Rugby	MVPA levels and self-determined motivation of boys and girls while participating in a prolonged unit (6–12 lessons) of the invasion game were determined by two instructional models (DIM and TGM)	RT3 Triaxial Accelerometry/ Self-determination questionnaire/ Enjoyment scale	Boys had significantly higher MVPA in rugby and football than controls, with no significant difference in motivation Girls had comparable levels of PA in football but significantly lower levels of PA activity in netball. There was also no significant difference in motivation
Harvey and Pill [40]		124; TGM Researchers/Teachers		QL: Interpretative analysis		To survey and explore scholars’ and teachers’ perceptions of TGM	Questionnair/ Twitter Chat/ Windschitl’s Dilemmas Framework	
Harvey, Smith [41]	US; TGM	174; Middle school students (7^th^ grade)/ Primary school students (4^th^ and 5^th^ grades)		QT: Non-experimental observational design	Basketball	Investigate the effect of TGM on MVPA/VPA and differences between gender/education levels	Accelerometry (Actigraph GT3xTriaxial)	TGM can help students accumulate VPA levels that meet national standardsMVPA/VPA levels are significantly higher for boys than for girls in both primary and lower middle school
Harvey and Atkinson [42]	US; TGM	1; Football coach		QL: Collaborative Action Research(CAR)	Football	Investigating the perceptions and experiences of a recreational soccer coach when integrating TGM into his coaching practice during an 8-state autumn training session	Model benchmarks/ PTRA tool/ Interviews	Coaches’ familiarity with the TGM affects model benchmark fidelity.The study demonstrated the benefits and challenges of incorporating the TGM into other novice youth sports coaching practices
Harvey and García-López [43]	US; TGM	174; Middle school students (7^th^ grade)/ Primary school students (4^th^ and 5^th^ grades)		QT: Quasi-experimental design	Basketball	Investigated whether students could accumulate MVPA recommendations in three-course contexts	Accelerometry (Actigraph GT3xTriaxial)	Middle and primary school students’ MVPA levels were significantly higher during play activities, but none of the students’ MVPA levels met the national physical activity recommendations
Harvey, Gil-Arias [31]	US; TGM	173; Middle school students (7^th^ grade)/ Primary school students (4^th^ and 5^th^ grades)		QT: Quasi-experimental pre-post-test design	Basketball	Investigate changes in need satisfaction and self-determined motivation between middle and primary school students in the TGM basketball unit	Model benchmarks/Need satisfaction and motivation questionnaire	Time significantly affected need satisfaction and autonomy in middle school and primary school students, but only on self-determination motivation in middle school students. Most of the time in middle and primary school programmes was spent in play. Teachers’ main behaviours were instruction, management, specific observation, corrective feedback and demonstration
Hodges, Wicke [30]	US; TGM	123; Middle school students (8^th^ grade)/ High school students (9^th^ grade, 12^th^ grade)		QT: Quasi-experimental design	Soccer/ Team Handball/ Football	Examine the effects of TGM on PA and GPP of secondary school students across schools, sports and age groups	TSAP/ Pedometers	TGM has a significant positive impact on middle school students’ GPP. Although it positively impacted students’ PA levels, it did not reach the nationally recommended levels
Williams and Hannon [44]	US; TGM	446 Middle school students		QT: Quasi-experimental design	Basketball/ Soccer/ Flag Football/ Volleyball	Comparison of PA levels of middle school students participating in co-educational and same-sex TGM physical education programmes in basketball, soccer, flag football and volleyball	Accelerometer	No significant differences were found in PA levels for male students across gender settings and sports units. No significant differences were found in the percentage of time spent on MVPA between female students in same-sex settings and male and female students in coeducational settings in the volleyball, flag football, and football units. Gender environment does not affect MVPA time for male students, but it does affect female students, depending on the sport. Male students spend more time on MVPA than female students in any gender setting and sports unit
Alagül and Gürsel [45]	TR; TGM	2; Teachers		QL: Participatory Action Research(PAR)	Net games	Discovering Physical Education Teachers’ Perspectives on Experiencing the Instructional Innovation Process Using the Tactical Games Model (TGM)	Interview	Teachers are expected to be actively involved in learning innovations in teaching models and to facilitate the adoption of innovations with the help of other teachers and experts
Garcia-Ceberino, Feu [46]	ES; TGAs/DIs	41; Primary school students (5^th^ grade)		QL	Football	Design and analysis of the similarities and differences between two homogeneous intervention programmes (i.e. Direct Instruction in Soccer “DIs”; Tactical Game Approach in Soccer “TGAs”)	SIATE Record Analysis	TGA can stimulate more engagement and motivation and better understanding and retention of what is being learnt in PE courses compared to DI
Gouveia, Gouveia [47]	PT; TGM/TOA	79; Middle school students (7^th^ grade, 8^th^ grade)		QT: Quasi-experimental non-equivalent control group pre-post-test experimental design	Invasion games: Soccer/ Handball/ Basketball	Compare the effects of TGA and TOA on students’ invasion game performance and explore whether the two teaching methods differ in motor engagement time (MET)	GPAI/ direct observation	Both instructional methods promoted off-ball and on-ball movement, and there were no significant differences in instructional effectiveness. However, students demonstrated better on-field decision-making and more vital on-ball decision-making skills during the invasion game unit TGM compared to the technique-oriented method. Moreover, TGM also provided students with more opportunities for motor engagement time (MET)
Güneş and Yılmaz [48]	TR; TGA/CA	60; High school students (9^th^ grade)		QT: Semi-experimental study	Basketball	Examine the effects of a tactical games approach to teaching and learning on students’ cognitive, affective, and psychomotor domain (ball handling, dribbling, passing, and shooting) skills and game performance (decision making, skill practice, support, game participation, and game performance) in real-life experiences in a basketball unit of a middle school physical education course	Basketbal Achievement Test/ Physical Education and Sports Attitude Scale/ Obsercation Scale/ GPAI	TGA and traditional teaching methods showed significant differences in post-test results in the cognitive and psychomotor domains, but no significant differences were found in the affective domain. At the same time, TGA showed a significant difference in the post-test results of all game performances. The TGA demonstrated significant differences in the post-test results for game performance’s decision-making and skill practice components. In contrast, no significant differences were reported in the post-test results for game performance’s support and participation components. There were no significant differences between the TGA and the TGA in the cognitive, affective, and psychomotor domains at the skill performance level. However, the TGA had significant differences in the decision-making, support, game participation, and game performance components of the game performance level. Overall, TGA is more suitable than traditional methods for improving game performance
Rodríguez-Negro, Falese [49]	ES; TGM/DIM	380; Primary school students (1^th^ grade– 6^th^ grade)		QT: Quasi-experimental design	Balance	Examine the effects of an 8-week DIM and TGM balance intervention programme on static and dynamic balance in primary school children	Anthropometric measurement/ Movement assessment battery for children-2 balance tests/ Standing stork tests	Both interventions improve students’ static and dynamic balance. DIM is more effective for students’ balance in the lower primary school age range. TGM may be more effective at higher ages
Coppola, Pignato [50]	IT; TGM/TS	66; Primary school students (4^th^ grade)		QT: Experimental studies/longitudinal studies Pre-post-post design	Invasion and Net/Wall game: Basketball/ Handball/ Football/Volleyball	Analyse the effects of two different teaching methods on the level of enjoyment of sport among nine-year-old pupils in the fourth year of primary school	16-item PACES(Physical Activity Enjoyment Scale) questionnaire	No significant difference was found between the two teaching methods on the level of enjoyment. However, the TGM group tended to be more positive on enjoyment than the TS group. In addition, the girls in the TGM group showed an increase in their scores on the post-intervention positivity scale
García-Ceberino, Antúnez [51]	ES; TGAs/DIs	41; Primary school students (5^th^ grade)		QT: Empirical investigations, quasi-experimental studies	Football	Quantify and compare the external (eTL) and internal (iTL) loads and the Ratings of Perceived Exertion (RPEs) generated by school football teaching programmes using two different teaching methods (TGA and DI) according to gender and teaching method	Inertial device(WIMU PRO)/ Borg’s RPE scale	TGA students reported higher iTL (heart rate) and performed more extended periods of high-intensity activity. Male students had higher eTL, iTL, and RPE values than female students. Both teaching methods increased the efficiency of students’ RPE. TGA was more beneficial to students’ fitness and health
García-Ceberino, Gamero [52]	ES; TGAs/DIs	41: Primary school students (5^th^ grade)		QT: Quasi-experimental longitudinal design	Footballl	Compare declarative and procedural knowledge levels after implementing two school football intervention programmes (one based on TGA and the other on DI) according to method, gender and experience (football practice)	Tactical Knowledge Assessment test in Football	Both teaching methods improved students’ declarative and procedural knowledge, and there was no significant difference. Boys had more experience and higher levels of knowledge compared to girls.TGAs will improve more with students who know about football
García-Ceberino, Gamero [53]	ES; TGA/DI	35; Primary school students (5^th^ grade)		QT: Quasi-experimental, longitudinal front-to-back design	Football	Examine and compare the differences in football technical and tactical learning outcomes of students using two different teaching methods	IMLP Foot Instrument	Both teaching methods improved students’ football skills and tactics, with no significant differences between groups. However, between the assessment tests, the learning level of the tactical programme was higher than that of the technical programme
Gonzalez-Espinosa, Antunez [54]	ES; TGA/DI	68: Primary school students		QT: Empirical design of quasi-experimental, pre-experimental and longitudinal operational strategies	Basketball	Describe and compare the physical demands (i.e., eTL and iTL) induced by two different teaching methods in the teaching of invasion games, such as school basketball, and determine if a relationship exists between eTL and iTL in assessment sessions and student performance indices in games	Instrument for the Measurement of Learning and Performance in Bsaketball(IMARB)/ Inertial recording device(Wimu)	Regarding external and internal loading variables, TGA achieved better results than DI, and TGA allowed the students to develop their physical fitness to a greater extent. Regarding performance in games, the students trained with TGA showed better performance indicators
González-Espinosa, García-Rubio [55]	ES; TGA/DI	70; Primary school students (5^th^ grade, 6^th^ grade)		QT: Quasi-experimental design	Basketball	External training loads under two different teaching methods were analysed and compared according to the game in physical education	Inertial device(WIMU)	TGA enables students to obtain higher external training load values than DI. At the same time, when the complexity of the game situation increases, the students’ external training load decreases. The intensity of the tasks provided by TGA can reach the physical activity goals recommended by the World Health Organisation to ensure the students’ health
Rodríguez-Negro and Yanci [56]	ES; TGA/DI	256; Primary school students (1^th^ grade– 6^th^ grade)		QT: Quasi-experimental design	Balance	Investigate the effects of two different teaching modes (DIM and TGM) on primary school students’ perceived physical strength, emotion, PA level, instructional time, active learning time and the relationship between instructional/active learning time, and analyse the differences in outcomes for students of different ages	Children’s OMNI scale of Perceived Exertion/ Feeling Scale electronic pedometer	There were significant differences between DIM and TGM in the relationship between perceived consumption, PA levels, instructional time, and instructional/active learning time. The PA levels of DIM were significantly higher among students in grades 3, 4, and 6
Sgrò, Barca [57]	IT; TGA/TOA	77; High school students		QT: Quasi-experimental study of a posttest design with a non-equivalent control group	Volleyball	Assess the impact of two different physical education teaching strategies on the affective domain of learning for high school students	Perceiver Motivational Climate in Sport Questionnaire/ Physical Activity Enjoyment Scale	TGM students scored higher on the enjoyment level and task engagement climate dimensions and lower than control students on the self-involvement climate dimension and negative enjoyment level.TGMs can provide valuable teaching and learning experiences that positively impact the affective domain of student learning
SgrÒ, Coppola [58]	IT; TGM	39; Primary school students (4^th^ grade)		QT: Quasi-experimental design	Volleyball	Assess the effect of the TGM teaching programme on the technical performance of volleyball among primary school students according to gender	TASP	Both boys and girls showed significant improvement in their volleyball skills with TGM instruction, while girls showed more improvement than boys
Gamero, Garcia-Ceberino [59]	ES; TGA/DI/STBU	49; Primary school students (6^th^ grade)		QT: Quasi-experimental design	Basketball	Quantify and compare the external (eTL) and internal (iTL) loads resulting from an intervention programme using three different teaching methods, TGA, DI and STBU, in teaching student basketball, based on the teaching methods and the student’s previous experiences	Inertial device(WIMU PRO)/ Borg’s RPE scale	The students using the TGA teaching method reported higher eTL and iTL values. Although students using the TGA instructional method were similar in heart rate levels to those using the other two, they spent more time on high-intensity activities, had more extended time in the running and sprinting speed ranges, and had better physical fitness. In addition, students with no basketball experience showed higher levels of maximum speed, while students with basketball experience had higher heart rate levels. So, TGA favours the physical condition and health of primary school students
Gamero, García-Ceberino [60]	ES; TGA/DI/STBU	55; Primary school students (6^th^ grade)		QT: Manipulated quasi-experimental longitudinal method	Basketball	Assess the acquisition of declarative and procedural knowledge by primary school students following the implementation of different basketball intervention programmes in a pedagogical context based on the pedagogical methods used and prior experience	Test of Declarative and Procedural Knowledge in Basketball(TDPKB)	All teaching methods increased the level of students’ declarative and procedural knowledge. At the same time, students who participated in the TGA programme gained higher levels of declarative knowledge. Students who had no prior exposure to basketball gained higher levels of knowledge through the TGA intervention programme
Gonzalez-Espinosa, Garcia-Rubio [61]	ES; TGA/DI	40; Primary school students		QT: Quasi-experimental design with randomised groups	Basketball	Analyse and compare students’ learning of the game of basketball under two different approaches	Basketball Learning and Performance Assessment Instrument(BALPAI)	Students in the TGA programme showed significant improvement on all 13 measured variables, whereas students in the DI programme showed improvement on only three variables. Therefore, the TGA is more applicable to basketball learning than the DI
Schembri, Coppola [62]	IT; TGM/TS	64; Primary school students (2^th^ grade)		QT: Quasi-experimental design	Handball	Assess the impact of different teaching programmes on the affective learning objectives (i.e. Enjoyment) of physical education lessons for primary school students	Physical Activity Enjoyment Scale	Students who participated in TGM had increased levels of enjoyment at the end of the course.TGM is an effective strategy that can support fun and motivating physical education courses
Sgrò, Coppola [63]	IT; TGM	39; Primary school students (4^th^ grade)		QT: Interpretive longitudinal studies	Volleyball	Evaluate the impact of a tactical games model teaching programme on primary school students’ volleyball performance	TASP	All students showed moderate to substantial improvements in their overall grades at the end of the intervention, and the progress was more excellent and more pronounced for the academically weak students than the academically strong students
Feu, Garcia-Rubio [64]	ES: TGM	23; Teachers		QL: Associative, comparative cross-sectional design	Handball	External load (eTL) in physical education courses was analysed according to the type of teacher (in-service or pre-service)	SIATE Record Analysis	The handball learning tasks designed and developed by in-service and pre-service teachers showed lower eTL values, while the TGM’s teaching plans had significantly higher eTL values
Garcia-Ceberino, Feu [65]	ES; TGA/DI	108; Primary school students		QT: Quasi-experimental design	Football/ Basketball	Students’ motivation was analysed according to four variables: students’ gender, the type of exercise taught, students’ experience and teaching methods, and the effect of students’ gender and experience on teaching methods was also analysed	Achievement Motivation in Physical Education test based on Atkinson’s theory(AMPET-e)	No significant difference was found between TGA and DI compared to each dimension. In football and basketball, male students perceived themselves as more athletic than female students. Experienced students were more competent in the sport than inexperienced students, making them less anxious and stressed about failure. Students who played football showed more commitment and perceived athletic ability than those who played basketball
Setiawan and Juliantine [66]	ID; TGM/NI	20; University students		Mix: QT; QL	Handball	Evaluate the impact of a tactical games model course on improving the formation of basic skills in men’s handball players	Shooting 、Pass、Dribbling Test/ In-depth interviews	TGM was significantly effective in improving the formation of basic skills in male handball players, and subjects offered positive and varied perceptions of TGM
Rodríguez-Negro and Yanci [67]	ES; TGM/DIM	168; Primary school students		QT: Quasi-experimental design	Balance	Investigate the effects of two different physical education intervention programmes on primary school students’ cognitive functions (i.e., creativity, attention and impulse control)	CREA test/ CARAS-R test	TGM is a successful instructional model that improves creativity and attention in elementary school students, and older (6th grade) students also improve impulse control during TGM sessions. In contrast, DIM did not observe significant differences in cognitive functioning in any of the analyses
Setiawan and Juliantine [66]	IT; TGM/DOA	67; Middle school students/ High school students		QT: Quasi-experimental study of pre-test and post-test designs with non-equivalent control groups	Volleyball	Assessing the impact of two physical education teaching strategies on promoting student improvement in volleyball competitions	GPAI	Students in the TGM programme showed significant improvement in support index and decision-making. Moreover, compared to the control group, there were improvements in decision-making, skill execution and support. It indicates that using TGM in high school physical education has improved students’ academic performance to some extent, provided a more accurate volleyball environment, and helped students better understand tactical awareness and achieve better athletic performance
Hartati, Ursa [68]	ID; TGM/NI	60; Middle school students		QT: Quasi-experimental design	Basketball	Determine the impact of tactical approaches in basketball learning on student learning creativity and motivation	Creativity questionnaire/ Student learning motivation questionnaire	Students who received the TGM intervention showed significant differences in the results of creativity and learning motivation in basketball learning. It shows that TGM can increase students’ creativity and learning motivation in basketball learning
Sucipto, Hambali [69]	ID; TGM/TA	56; Middle school students		QT: Experimental research	Physical Education Course	Explore the impact of learning methods and gender on students’ enjoyment of sport	Physical Activity Enjoyment Scale Questionnaire(PACES)	Both learning methods showed significant effects on students’ enjoyment of learning. Regarding gender, both genders significantly affect students’ enjoyment of learning. However, there is no interaction between gender and learning methods in affecting students’ enjoyment. The tactical approach has a more significant effect on students’ enjoyment of sports learning than the technical approach. Male students had a more significant effect on developing learning enjoyment in sports than female students

GB: United Kingdom; US: United States; TR; Turkey; ES: Spain; PT; Portugal; IT: Italy; ID; Indonesia; TGA: Tactical Games Approach; TGM: Tactical Games Model; DIM: Direct Instructional Model; DI: Direct Instuction; TOA: Technique-Oriented Approach; CA: Conventional Approach; STBU: Service Teacher’s Basketball Unit; TGAs: Tactical Games Approach to Soccer; Dis: Direct Instruction in Soccer; TS: Teaching Skill; NI: None-Intervention; TA: Technical Approach; Mix: Mix-methods; QT: Quantitative; QL: Qualitative; GPAI: Game Performance Assessment Instrument; TASP: Team sport Assessment Procedue.

## 3 Results

The study’s results reported that 38 studies globally fulfilled the criteria for this review to be included in the systematic review. In the next section, we will detail the current status and main findings of the tactical games model in physical education and discuss them in light of the issues of this review.

### 3.1 Background of the study

**Fig 2:**
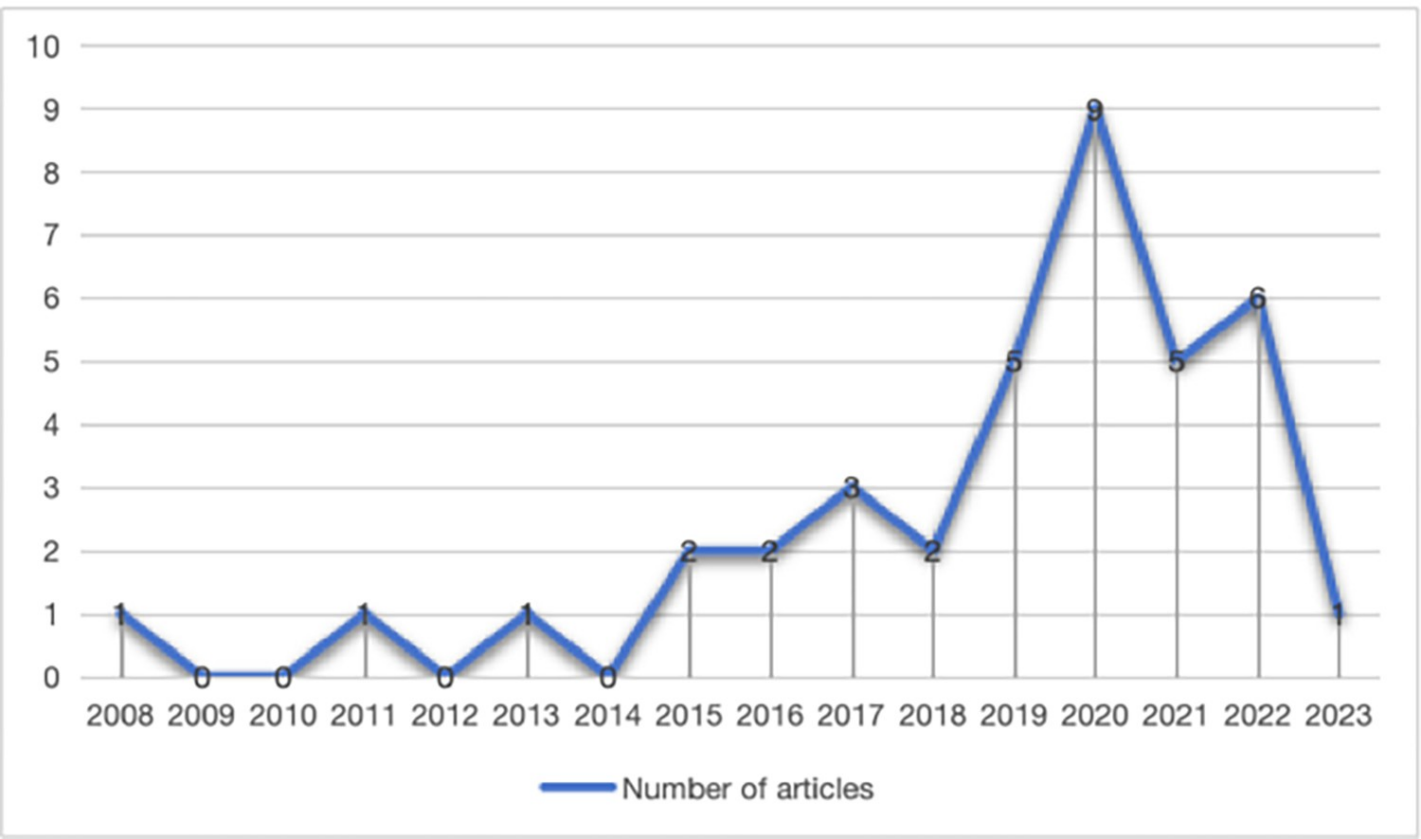
A graphical representation of the publication trends in the literature on Tactical Games Model.

Firstly, in terms of the year of publication, the first article on the tactical games model was published in 2008. However, research on TGM came to a standstill, and in the following six years, only one relevant article was published in 2011 and 2013, and there was no literature on TGM published in 2009, 2010, 2014 and 2015. It was only at the beginning of 2015 that researchers gradually showed interest in the study of TGM. It was not until 2020 that research on TGM peaked, with nine publications in the current year. Although this review ended in January 2024, only one published literature in 2023 was included in this study, probably due to the article publication process. A graphical representation of the publication trends in the literature on Tactical Games Model is shown in Fig 2.

According to the results of the current study, the highest number of studies using both instructional modalities for intervention accounted for 55.3% of all the literature (n = 21). At the same time, 11 papers used TGM alone as an intervention. The rest of the literature includes two studies that used all three instructional models for interventions, two that compared TGM and no intervention, and two that did not mention interventions. Furthermore, the country in which most of these studies on TGM were conducted was Spain, accounting for 36.8% of all literature (n = 14), followed by the United States (n = 7) and Italy (n = 6), with the rest of the studies being conducted in countries such as the United Kingdom, Indonesia, Turkey and Portugal, respectively. There was also one piece of literature that did not mention the country where the study was conducted.

### 3.2 Participants

The results of this study report a total of 3,357 subjects who participated in the studies in all 38 articles. The number of participants in these studies ranged from 1 to 446. The majority of these participants were students (88.4%), with 24 (55.8%) studies examining students at the primary school level, 13 (30.2%) studies examining students at the secondary school level (both middle school and high school levels), only three studies focusing on teachers, and one each of the other literature examining college students, coaches, and researchers.

### 3.3 Types of research and data collection instruments

From the 38 articles in this review, 29 used quantitative methods (76.3%), seven articles used qualitative methods (18.4%), and only two articles used mixed methods (5.3%). In these quantitative studies, several data collection instruments were used, among which the most used data collection instruments were questionnaires such as a Self-determination questionnaire, Enjoyment scale, Need satisfaction and motivation questionnaire, Physical Education and Sports Attitude Scale, Observation Scale and PACES (Physical Activity Enjoyment Scale) questionnaire (n = 11). As well as PA and load measurement instruments such as RT3 triaxial accelerometry, Actigraph GT3XTriaxial, Pedometers and inertial device (WIMU PRO) (n = 11), followed by game performance assessment instruments such as the Game Performance Assessment Instrument (GPAI) and the Team sport Assessment Procedure (TSAP) (n = 8), and finally test instruments such as the Standing stork test, Tactical Knowledge Assessment test and CARAS-R test (n = 7). Data collection instruments such as group and individual interviews, observation and monitoring assessments and questionnaires were used in the qualitative studies. Finally, in the two mixed-methods studies, data collection was used as described above in both quantitative and qualitative: GPAI, tests and interviews.

### 3.4 Domains of application

Concerning the domains in which the TGM has been used in the field of physical education, the present study revealed that the invasion game unit was the most used domain, with basketball being the most popular sport (n = 14), followed by football (n = 11), handball (n = 6), rugby (n = 4) and hockey (n = 1). Similarly, several studies have applied the TGM to the net/wall game unit, with six papers focusing on volleyball and one each on tennis and speed badminton. In addition to this, three studies focused on balance programmes. One study applied the TGM in a physical education course. Only one article did not mention a specific area of physical education.

### 3.5 Purpose of the study

This study’s findings indicate that most of the literature is not focused on one topic alone but instead has multiple research topics and objectives that outnumber the 38 publications included in the review. So, this review has grouped these topics of research on the Tactical Games Model into seven distinct subjects: (1) Twenty-one articles compared the TGM to other teaching methods, a topic that has been of most interest to many researchers on the TGM;(2) Physical Activity Levels, Fitness Levels, and Sports Loads of Students, with 12 articles researching this topic;(3) Ten articles focused on the affective domain of students, including aspects of students’ self-determined motivation, need satisfaction, enjoyment of physical education and enjoyment of physical education courses;(4) The level of students’ game performance, skills and knowledge acquisition of the participating sports, there were 13 articles on this topic, with the majority of the researchers focusing on the level of skills;(5) Perceptions and attitudes of researchers, teachers, coaches and students towards TGM in the field of physical education, with four articles devoted to this topic;(6) Only two articles examined the topic of students’ sport participation time; (7) Students’ cognitive functions (creativity, attention, impulse control), also only two articles examined this topic.

### 3.6 Research results

As mentioned above, most literature focuses on more than one research topic. Therefore, the review results will be summarised in this section according to the seven topics mentioned above.

Firstly, in comparing TGM and other teaching methods, the results of this review report that TGM is a successful teaching model [67]. These teaching models used for comparison include Direct Instructional Model (DIM), Direct Instuction, Technique-Oriented Approach, Conventional Approach, Service Teacher ’s Basketball Unit, Teaching Skill, Technical Approach, and None-Intervention. However, no research suggests that TGM is necessarily better than other teaching methods. Among the 21 articles in the literature, 14 articles (66.7%) explicitly reported more positive impacts of TGM compared to other teaching methods. However, there were also 7 articles (33.3%) whose findings indicated that TGM was not significantly superior to other teaching models. Additionally, students who experienced the TGM programme demonstrated significant improvements in the following areas: Levels of MVPA and VPA; Course Participation; Motor Engagement Time (MET); Cognitive Competence; Physical Fitness Levels; Internal and External Loads (ITL, eTL); Affective domain of learning; Basketball learning; Creativity; Attention; and Decision-Making Performance. Moreover, compared to other teaching models, Enjoyment of Learning, Motivation, Level of Football Skills and Tactics, Level of Football Knowledge, Level of Enjoyment, Balance, Effectiveness of Ball Teaching, and Level of PA did not significantly improve. Moreover, TGM and other teaching methods were able to have a positive impact on students in the topics of Off- and On-ball Movement [47], Cognitive and Psychomotor Domains [48], Balance [49], Level of Declarative and Procedural Knowledge [52,60], Level of Football Technique and Tactics [54], and Enjoyment of Learning [69].

Of the 12 articles focusing on students’ physical activity levels, fitness levels, and motor loads, 9 articles (75%) reported that TGM could improve students’ performance positively [32,37,41,51,54–56,59,64]. However, results from 5 articles showed that while TGM improved students’ PA levels, it did not meet the national physical activity recommendations [39,43,44,59]. In comparison with other teaching methods, articles have reported that the level of physical activity of students participating in TGM programmes is not better than that of students participating in programmes with other teaching methods [39,44,56].

Most studies have shown that TGM improves students’ affective domains (self-determined motivation, need satisfaction, enjoyment of physical education and enjoyment of physical education programmes). Seven articles (70%) demonstrated TGM as an effective strategy for supporting interesting and motivating physical education courses, stimulating students’ interest in physical education courses and increasing motivation to learn [62]. However, there are still some articles whose results show that TGM does not significantly improve students’ affective domains [48]. However, TGM had a more positive impact on enjoyment level [50] and enjoyment of physical education and learning [69] than other teaching methods.

All studies on students’ game performance levels and technical and tactical knowledge acquisition of participating sports reported that TGM can positively impact students. In particular, game performance [63], game decision-making and decision-making competence [47,48], and tactical awareness were able to improve student performance better compared to other teaching methods. However, some articles show that differences between genders [52,58] and ages [49] also affect students’ results in this topic.

Positive perspectives on TGM implementation were reported in articles exploring the perceptions and attitudes of researchers, teachers, coaches, and students toward TGM. Some studies have shown that TGM enhances students’ physical fitness, allows them to enjoy new games, and is a new method to help students learn to exercise throughout their lives [37]. Some studies have also reported that the TGM teaching method stimulates players’ interest and participation in training and increases their motivation [38]. Some studies even suggest that teachers should be actively involved in the innovation of teaching models, as the positive impact of TGM can facilitate the adoption of innovative teaching methods [45]. In addition to the above topics, there are no accurate results for the time students spend participating in sports in PE lessons. However, TGM can provide students with more Motor Engagement Time (MET) and increase their time actively learning. In addition to the above topics, there were no accurate results on students’ time participating in PE lessons. Although TGM provided students with more motor engagement time (MET) and increased their time for active learning, there were no significant differences compared to other teaching methods [47,56]. However, within the domain of students’ cognitive functioning (creativity, attention, and impulse control), studies have reported TGM as a successful instructional model because TGM not only improves students’ creativity and attention but also improves their impulse control and shows superior improvements compared to other instructional methods [67,68].

## 4 Discussion

This systematic review aimed to investigate the results of research conducted on the Tactical Games Model (TGM) in physical education. The 38 articles screened from the four databases were analysed inductively and grouped according to the six dimensions mentioned above: study background, participants, type of study and data collection instruments, field of application, purpose of the study, and study results.

Researchers have not focused on the TGM in the field of physical education since 1997 [27] when a simplification of the Teaching Games for Understanding (TGFU) approach was proposed as the Tactical Games Model (TGM), with only three articles examining the TGM in the field of physical education prior to 2015.TGFU is also mentioned in some reviews with information on game-centred teaching methods as still being the most talked about game-centred teaching method in physical education [71,72]. In contrast, researchers began to show interest in the TGM after it was redefined and improved in 2013 [73]. More and more research on TGM is being conducted in physical education, broadly in line with the results reported in several systematic reviews [23,74]. Although TGFU is still a Hot Topic of research, TGM has gradually become a game-centred teaching method that is second only to TGFU in the attention of researchers. This also reflects that TGM is gradually being recognised by researchers in the field of physical education and indirectly indicates the need to improve teaching effectiveness in physical education, which precisely demonstrates that TGM can satisfy this need of researchers in some aspects. Even though more and more researchers have been conducting studies on TGM in recent years, the paucity of articles compared to studies on TGFU still suggests that tactical games model are still in their infancy in the field of physical education so that future researchers could conduct more studies on TGM.

Furthermore, the results of the current study reported that in half of these studies focusing on TGM, the articles compared TGM with other types of teaching methods in their studies to validate the effectiveness of TGM in teaching and learning. This follows the majority of studies reported by [72] in their systematic review of GCAs, still comparing the results of two different teaching methods. However, this way of comparing different teaching methods in an attempt to verify that one is better than another has been recognised by researchers as undesirable, and such an approach does not necessarily identify the merits of the teaching method [75]. However, researchers now widely recognise practice-referenced research as providing a better study of student mastery and learning outcomes in sports [23,76]. Nevertheless, in this study, only 11 articles conducted intervention-focused studies using TGM alone, and these studies can be more intuitive about the effects of TGM on students in physical education. They can find the effects of TGM on students in different domains, such as psychological, affective, and cognitive. Therefore, researchers can conduct more TGM-focused intervention studies in the future. In addition, although the TGM was adapted and simplified by researchers in the United States, the country with the most research on the TGM is Spain, where 14 articles have been conducted. It has been suggested in previous systematic reviews on GCAs that an increasing number of researchers from Europe are conducting studies on GCAs [23,72].

Moreover, the current review confirms that European researchers are the mainstay of researching TGM, with 27 (71%) of the articles included in this review being European studies. Studies are also conducted in Southeast Asia [66,68,69]. While most research on TGM has been conducted in Europe and the Americas, researchers in Asia are beginning to pay attention to TGM. The positive attitudes of these researchers towards TGM will help us to understand the effects of TGM in physical education in different cultures. They will allow future researchers to have the confidence to develop TGM in the educational systems of different countries to increase the experience of the effects of TGM on education in new cultural and social contexts. Experiences of the impact of TGM on education in new cultural and social contexts.

Significant differences regarding the participants exist in the articles included in this review. First, the number of participants ranged from 1–446. With the lowest number of participants in the studies of [42,45], [42]investigated a football coach’s perceptions and experiences when incorporating the TGM into his training, whilst [45] investigated the perceptions of two physical education teachers about the process of pedagogical innovations following the use of the TGM. Typically, most articles have between 20–77 participants, and this is because in most of these articles, the researcher investigated students at one level of education, and the participants were mostly from classes set up in the school. The study usually selects more than one class to randomly assign to experimental and control groups and conduct the study simultaneously. However, the study results reported that the number of participants with 10 articles ranged from 108–446. These articles have many participants because each study focused on students at multiple educational levels. For example, [49] investigated six grades of primary school students to compare the effects of TGM and DIM on students of different ages with balanced abilities [56], on the other hand, investigated primary school students between the ages of 6 and 12 in a study exploring whether TGM and DIM have different effects on PA levels in different grades. Although the most significant number of participants in the [44] study selected for this review was a whopping 446, there was no comparison of the differences between students in different grades, but rather a focus on the differences between the genders of students participating in the TGM programme. In addition to this, the results of the current study found that the majority of articles (n = 37) examining TGMs in the field of physical education focused on K-12 students, a result that is different from the ideas presented by review articles focusing on other instructional models [74,77]. Whereas half of the articles in this review reported that the study population was primary school students (n = 24), only 13 investigated secondary school students (both middle school and high school levels). This result differs from those presented in previously conducted review articles on GCAs that suggested that secondary school students were the primary study population [23,72]. This suggests that researchers have recognised that developing an interest in physical learning in younger children is crucial and that developing children’s interest in sports at an early age reduces their abandonment of participation in sports as they grow up [78]. At the same time, game-centred teaching methods similar to TGM do help teachers improve their teaching and learning in physical education courses, a result that is also in line with the suggestion by [72] that more attention should be paid to the use of GCAs in younger children.

Nevertheless, this review also identified a lack of research investigating TGM in higher education, and although [73] recommended the use of TGM in K-12, preparing students for lifelong participation in physical activity is an integral pedagogical goal at all levels of education [79,80]. Some studies have implemented TGM in higher education and reported positive results; TGM does enhance university students’ mastery of handball skills, and those university students who participated in the study expressed interest and positive attitudes towards TGM [66]. Therefore, in the future, TGM research can be conducted at more educational levels, significantly higher education, to investigate whether TGM can impact university students. In addition, only three articles were conducted with teachers as the subjects of the study, which could be due to various reasons. The selection of teachers as the study subjects may be a challenge for the researcher as there are a limited number of teachers in the school. Unlike students, a sufficient number of samples can be obtained for quantitative research.

Meanwhile, teachers may be more selective in using their familiar teaching methods and less willing to embrace new teaching methods. There may be many problems contributing to the lack of TGM research focussing on teacher populations within the field of physical education. Nevertheless, it has been shown in many studies that these participating PE teachers have expressed positive perceptions of the TGM and that many PE teachers are willing to actively participate in the innovation of the pedagogical model [40,45]. Therefore, future research on TGM in physical education could be more about the teacher population.

Most articles included in this systematic review used quantitative research methods (76.3%). This result aligns with those presented in the systematic review on GCAs conducted by [72].The implementation of quantitative research methods to analyse TGM in the field of physical education can help researchers to visualise and analyse the pedagogical effects of courses adopting TGMs in a more intuitive way through the data, such as the effects of developing students’ motor skill levels [53,58], the effect of enhancing students’ game performance [30,63], the effect of improving students’ PA levels [32,41,44], the effect of enhancing students’ motivation to learn in sport [31,68], among other things. A wide variety of data collection instruments were used in these studies employing quantitative research methods, with the majority of articles using questionnaire scales (n = 11) (e.g. Self-determination questionnaire [39]; Enjoyment Scale [50,57]; Need satisfaction and Motivation questionnaire [31]; Physical Education and Sports Attitude Scale [48]; Feeling Scale [56]; Physical Activity Enjoyment Scale Questionnaire [69], etc.) and assessment of PA levels and exercise load measurement instruments (n = 11) (e.g. RT3 triaxial accelerometry [32,39]; Actigraph GT3XTriaxial [41,43]; Pedometers [30,56] and inertial device (WIMU PRO) [51,59], among others).In contrast, there was no excessive variety of in-game performance assessment instruments (n = 7), with researchers generally opting for the more widely used GPAI [29,47] and the TSAP [58,63]. Besides the above data collection instruments, this review identified instruments not reported in previously conducted systematic reviews of game-centred pedagogical approaches. There are only a few articles that have used tests to collect data about skill and knowledge levels in sports (n = 7) (e.g., Basketball Achievement Test [48]; Standing Stork Test [49]; the Tactical Knowledge Assessment Test [52], etc.).This study found that from these articles that used quantitative research methods, it was found that researchers are currently focusing more on assessing the impact of TGM on students’ psychological and emotional domains and physical activity levels without focusing on analysing students’ game performance and skill acquisition, which is in line with the proposal made by [81] in 2018 that in Physical Education the learning of technical and tactical learning of sports events that the assessment of the situation is the focus of the investigations of teachers and researchers. This situation may be caused by the social environment, especially after the Covid-19 pandemic, enhancing students’ interest in physical education programmes and improving their physical condition to ensure their health would be a priority in the field of physical education research [82].

Furthermore, in the future, researchers can increase the studies on students’ game performance and technical and tactical mastery so that students can learn more about the sport disease, participate in it and get pleasure from the game, which may also allow students to participate in physical activity actively. Thus, they may improve their physical health as well. In addition to the articles that used quantitative research methods, seven of the articles included in this study used qualitative research methods (18.4%). These studies used methods such as group and individual interviews [38,45], observational and monitoring assessments [37], and questionnaires [40] to collect data. Using qualitative research methods can help us understand the perceptions and attitudes of teachers, students, and coaches towards TGM. It can also provide more insight into the attitudes and opinions of researchers in physical education by analysing previous knowledge and experiences of TGM.

Additionally, only two articles used a mixed-methods approach to study TGM [36,66], using the same data collection instruments as in the quantitative and qualitative research methods. Although most of the articles adopted quantitative research methods, conducting research on TGM in the field of physical education cannot be limited to one research approach, and the researcher should be flexible in choosing the appropriate research approach according to the research objectives to obtain the best research results. As suggested by [74] in 2020, researchers can better analyse the effects of GCAs through the flexible use of different research methods.

Regarding the 38 articles in this review, most chose to study TGM in the invasion unit. Basketball was highly preferred by the researchers (n = 14), followed by football (n = 11). Furthermore, these types of competitive sports develop participants’ tactical and technical literacy, which is to some extent more in line with the pedagogical philosophy of TGM, and the application of TGM in these sports can fully utilise its role in setting up tactical problems [73]. Additionally, researchers have preferred to study TGM in team sports such as handball [62,66], rugby [38], hockey [32] and volleyball [58,63] to study TGM in team sports. This indicates that implementing TGM in physical education is transferable from one game to another and can be implemented in many different sports. Besides studying TGM in invasion game units, researchers have also applied TGM in net/wall game units such as tennis [39] and speedminton [37], in addition to articles on TGM in balance programme (n = 3). This study found that researchers preferred to research TGM in team sports (e.g., basketball, football, volleyball, and rugby).

In contrast, fewer studies focused on TGM in individual sports, a result that is consistent with those described in the systematic review on GCAs conducted by [72,83]. However, a single focus on team sports in a limited number of sports does not enrich the research on TGM in physical education. Future researchers could research TGM in more sports, such as individual sports like badminton and table tennis. Focusing on a broader range of sports could help develop TGM. It could also help students improve their physical health and increase their motivation to participate in physical activity.

The majority of these articles on TGM in the field of physical education compared TGM with other teaching methods, with the traditional "direct instruction model" being the teaching method most frequently used for comparison (85.7%) [32,39,49,65,67,69], as well as the Technique-Oriented Approach [47,57] and Conventional Approach [48], among other pedagogical approaches. The fact that traditional teaching methods are given too much attention indicates that new teaching methods are still not recognised by teachers in the current field of physical education, and they are still used to using the direct teaching method in their daily teaching, which is not very effective in helping to improve the effectiveness of physical education teaching. In addition to conducting comparisons between TGM and other teaching methods, researchers have made it more important to enhance students’ physical learning outcomes as a goal of the study, such as physical activity levels [32,41,43,44], sport load [51,59], game performance [29,47,48,63], skill level [49,53,58,66], and knowledge acquisition level [52,60]. This result is to the pedagogical objectives of physical education in schools, through which students can acquire specific motor skills and improve their physical fitness [84,85]. Furthermore more and more articles are beginning to focus on students’ psychological and emotional domains such as self-determined motivation [31,39,68], need satisfaction [31], enjoyment of physical education learning [50,69] and enjoyment of PE programmes [57,62]. Some researchers have suggested that the influence of intrinsic factors such as motivation and satisfaction with learning needs on students should not be ignored, as these factors may extend to some extent into students’ lives during the learning process [86,87]. So, there is a definite need to increase research in physical education in the future on psychological and emotional domains such as motivation, satisfaction with learning needs, and so on.

Besides the above domains that have received more attention, the current study also found some articles that examined TGM in the context of students’ cognitive functioning and motor participation time, which suggests that researchers have a positive attitude towards the pedagogical effects of TGM and believe that TGM may have an impact on students’ cognitive abilities [67,68]. Moreover, students may participate in more sports during the TGM programme [47,56]. Only four articles explored the perceptions and attitudes of teachers, researchers and students, among others, towards TGM. Focusing on the attitudes and perceptions of teachers and researchers, among others, towards TGM can help to understand their experiences of using and researching TGM. It can contribute to the development of TGM in Physical Education. One of the apparent advantages of this is that it may be possible that by analysing the perceptions and attitudes of teachers towards TGM, it will be possible to understand the reasons for the teachers’ current preference for the use of the direct method of teaching and learning, and that it may be possible to adopt ways of making the new method of teaching accessible to the teachers in the future and to use it in everyday teaching and learning. Therefore, in the future, researchers can investigate TGM from various fields so that TGM can be better developed in physical education and be used more in daily teaching.

As mentioned above, this study found that most articles compared TGM and other instructional models. However, no articles reported that TGM was significantly superior to other instructional models in terms of pedagogy. [52] conducted an intervention with the Tactical Games Approach in a football course on a primary school campus, and the results showed that both instructional methods enhanced students’ declarative and procedural knowledge. However, the Tactical Games Approach did not show a significant superiority over the Direct Instruction method. The same results were reported in the study of [65], where the tactical games approach did not show superiority over the direct instruction approach in all dimensions of students’ motivation to learn. However, most of the articles still showed that TGM positively impacted some topics more than other teaching methods. For example, [67] suggest that TGM is a successful instructional model that effectively improves the creativity and attention of elementary school students. In contrast, the direct instruction method does not show improvement. The results of the study by [66] also reported that TGM was effective in improving the basic skills of men’s handball players, and these players also agreed that TGM was a very suitable model of instruction for training and teaching and that they were more willing to participate in TGM than in traditional teaching methods.This review found that although most articles compared TGM with other instructional models, the focus was on different topics, and the results examined differed. Whilst most researchers have reported positive results of TGM in physical education, not all topics are positively impacted compared to other teaching methods. This result differs from those presented in previous review articles on other teaching methods [23,72,74,77]. At the same time, there are some articles reporting that TGM did not have a positive impact on some of the topics. For example, [48] focused on students’ affective domain in a basketball programme, whereas TGM did not significantly improve the emotional domain. For students’ PA levels, although all PA levels increased after the intervention of the teaching method, there are still articles reporting that such results did not reach the national recommended PA levels [30,43].

In addition to the above, most articles that have examined TGM in physical education have reported positive results across various topics, suggesting that TGM is indeed a pedagogical approach that can improve teaching and learning outcomes. Most of these studies focus on K-12 students; however, there is a lack of research on teachers and higher education. Particularly in higher education, students’ physical health, motivation, and decision-making competence have also been the focus of attention. This review also found that the findings of many articles show the positive impact that TGM can have on students’ decision-making. Despite the lack of research focusing on TGM in higher education, it has been suggested that TGM can be effective in improving the skills of collegiate handball players and that these players recognise the pedagogical effects of TGM [66]. In the future, researchers can focus on the effects of TGM in higher education to help university students improve their physical health, enhance their motivation to study and train them to participate actively in physical activity. It is also essential to assess whether the improved effects of TGM on decision-making can impact students in higher education, helping them improve their skills in cognitive domains.

## 5 Limitation

Although this study used a systematic review methodology and set appropriate eligibility criteria to analyse the results entirely, there are still some limitations of this systematic review. This review selected four high-impact online databases, whereas the results might have differed slightly if other databases had been included. Secondly, this review limited the language in which the articles were written to English, and many articles use other languages to study TGM in the field of physical education, so further research should cover more databases and literature in other languages. Meanwhile, this review analysed TGM-related articles within the field of physical education and found that most articles were researched in a school context. Moreover, future research should explore the effectiveness of TGM in various educational contexts, not only in the school context but also in other contexts, such as professional sports teams or extracurricular training organisations, so that TGM can be widely applied in physical education to help students to improve all aspects of learning.

## 6 Conclusion

This systematic review of Tactical Games Model reports on the characteristics of TGM research in physical education and analyses the relationship between these characteristics and research. In the results of the analyses, firstly, although many researchers have focused on TGM, there are many publications on TGM. However, there are still differences in the definition of the Tactical Games Model, and many teachers and researchers still do not distinguish the Tactical Games Model from "Teaching Game for Understanding" (TGFU). In the future, researchers still need to clarify the significance and impact of the Tactical Games Model in Physical Education so that a broader range of people can understand the TGM.

Furthermore, following our review, there is still a lack of high-quality research. These results suggest that research on the Tactical Games Model is still in its infancy, that there is a lack of empirical research on it in a variety of sports, and that there is still a need for teachers and researchers to conduct extensive research on the definition of the Tactical Games Model and its implementation in out-of-school settings in order to validate the effectiveness of the Tactical Games Model and to improve instructional programmes even though in recent years some articles have begun to develop research on TGM in the field of physical education and have explored the effects of TGM in different topics in the psychological, affective, cognitive and motor domains. Meanwhile, most articles focus on K-12 students, and there is a lack of research on teachers, coaches, and higher education. Moreover, the invasion game unit is the sport favoured by these researchers, and the effects of TGM are transferable across game units [73]. These results suggest that research on the Tactical Games Model is still in its infancy, that there is a lack of empirical research on it in a variety of sports, and that there is still a need for teachers and researchers to conduct extensive research on the definition of the Tactical Games Model and its implementation in out-of-school settings in order to validate the effectiveness of the Tactical Games Model and to improve instructional programmes.

According to the results of the analysis, most of the studies compared TGM with the Direct Instruction Model (DIM), a skill-centred teaching methodology currently used in teaching physical education in schools. While TGMs are more effective than DIMs in some domains, different instructional models have unique benefits. Researchers can compare TGM with different instructional models in the future through different research methods, such as qualitative and mixed methods. On the other hand, although TGM is a very effective teaching and learning model for students and its implementation in physical education is also effective in improving aspects such as motivation, enjoyment, fitness level and skill level of students in certain sports, various factors in the field of education such as cognitive, affective and social factors are essential in actual teaching and learning. Therefore, it also provides exciting research directions for subsequent researchers.

## Supporting information

S1 File PRISMA 2020 checklist. (DOC)

S2 File All studies identified in the literature search. (XLSX)

## References

[1] Tomanek M, Lis A. Managing information on the physical education research field: Bibliometric analysis. Physical education of students. 2020;24(4):213–26.

[2] Aktop A, Karahan N. Physical education teacher’s views of effective teaching methods in physical education. Procedia-Social and Behavioral Sciences. 2012;46:1910–3.

[3] Wyant J, Baek J-H. Re-thinking technology adoption in physical education. Curriculum Studies in Health and Physical Education. 2019;10(1):3–17.

[4] Fernandez-Rio J, de las Heras E, González T, Trillo V, Palomares J. Gamification and physical education. Viability and preliminary views from students and teachers. Physical Education and Sport Pedagogy. 2020;25(5):509–24.

[5] Hinojo Lucena FJ, López Belmonte J, Fuentes Cabrera A, Trujillo Torres JM, Pozo Sánchez S. Academic effects of the use of flipped learning in physical education. International journal of environmental research and public health. 2020;17(1):276. 10.3390/ijerph17010276 PMC6981672 31906054

[6] Cowley JG, McIntosh I, Kiely J, Collins DJ. The post 16 gap: how do young people conceptualise PE? An exploration of the barriers to participation in physical education, physical activity and sport in senior school pupils. International Journal of Adolescent Medicine and Health. 2021;33(6):313–21. doi: 10.1515/ijamh-2021-0003 34187138

[7] Abad Robles MT, Collado-Mateo D, Fernandez-Espinola C, Castillo Viera E, Gimenez Fuentes-Guerra FJ. Effects of Teaching Games on Decision Making and Skill Execution: A Systematic Review and Meta-Analysis. Int J Environ Res Public Health. 2020;17(2):505. doi: 10.3390/ijerph17020505 31941138 PMC7013807

[8] Farrow D, Pyne D, Gabbett T. Skill and physiological demands of open and closed training drills in Australian football. International Journal of Sports Science & Coaching. 2008;3(4):489–99.

[9] Kagan S. Excellence and equity. Kagan Online Magazine. 2009.

[10] Raiola G, Tafuri D. Teaching method of physical education and sports by prescriptive or heuristic learning. Journal of Human Sport and Exercise. 2015;10(1):S377–S84.

[11] Blomqvist M, Luhtanen P, Laakso 1 L. Comparison of two types of instruction in badminton. European journal of physical education. 2001;6(2):139–55.

[12] Casey A, Valenzuela AV, Giménez AM. What are we being told about how to teach games? A three-dimensional analysis of comparative research into different instructional studies in Physical Education and School Sports. RICYDE Revista Internacional de Ciencias del Deporte. 2010;6(18):37–56.

[13] Thorpe R, Bunker D. Issues that arise when preparing to teaching for understanding. Bulletin of Physical Education. 1983;19(1):9–11.

[14] Turner A, Martinek TJ. Teaching for understanding: A model for improving decision making during game play. Quest. 1995;47(1):44–63.

[15] Hewitt M, Edwards K, Pill S, editors. Teaching styles of Australian junior tennis coaches. 2015 Game Sense for Teaching and Coaching Conference: Conference Proceedings; 2016: University of Canterbury, School of Sport and Physical Education.

[16] Hopper T. Teaching games for understanding: The importance of student emphasis over content emphasis. Journal of Physical Education, Recreation & Dance. 2002;73(7):44–8.

[17] Holt JE, Ward P, Wallhead TL. The transfer of learning from play practices to game play in young adult soccer players. Physical Education and Sport Pedagogy. 2006;11(2):101–18.

[18] Kirk D. Physical education futures: Routledge; 2009.

[19] Haerens L, Kirk D, Cardon G, De Bourdeaudhuij I. Toward the development of a pedagogical model for health-based physical education. Quest. 2011;63(3):321–38.

[20] Kirk D. Educational value and models-based practice in physical education. Educational Philosophy and Theory. 2013;45(9):973–86.

[21] Metzler M. Instructional models in physical education: Routledge; 2017.

[22] Bunker D, Thorpe R. A model for the teaching of games in secondary schools. Bulletin of physical education. 1982;18(1):5–8.

[23] Harvey S, Jarrett K. A review of the game-centred approaches to teaching and coaching literature since 2006. Physical Education and Sport Pedagogy. 2014;19(3):278–300.

[24] Light R, Tan S. Culture, embodied experience and teachers’ development of TGfU in Australia and Singapore. European Physical Education Review. 2006;12(1):99–117.

[25] Singleton E. More than" Just a Game": History, Pedagogy, and Games in Physical Education. Physical & Health Education Journal. 2010;76(2).

[26] Mitchell S, Mitchell SA, Oslin J, Griffin LL. Teaching sport concepts and skills: A tactical games approach: Human Kinetics Publishers; 2020.

[27] Griffin LL, Mitchell SA, Oslin JL. Teaching sports concepts and skills: A tactical games approach: Human Kinetics Publishers (UK) Ltd; 1997.

[28] Oslin JL, Griffin LL, Mitchell SA. Teaching sport concepts and skills: A tactical games approach: Human kinetics; 2006.

[29] Sgrò F, Barca M, Schembri R, Coppola R, Lipoma M. Effects of different teaching strategies on students’ psychomotor learning outcomes during volleyball lessons. Sport Sciences for Health. 2022;18(2):579–87.

[30] Hodges M, Wicke J, Flores-Marti I. Tactical Games Model and Its Effects on Student Physical Activity and Gameplay Performance in Secondary Physical Education. Physical Educator. 2018;75(1):99–115.

[31] Harvey S, Gil-Arias A, Smith ML, Smith LR. Middle and Elementary School Students’ Changes in Self-Determined Motivation in a Basketball Unit Taught using the Tactical Games Model. Journal of Human Kinetics. 2017;59(1):39–53. 29134047 10.1515/hukin-2017-0146 PMC5680685

[32] Harvey S, Smith L, Fairclough S, Savory L, Kerr C. Investigation of Pupils’ Levels of MVPA and VPA During Physical Education Units Focused on Direct Instruction and Tactical Games Models. Physical Educator-Us. 2015;72:40–58.

[33] Page MJ, McKenzie JE, Bossuyt PM, Boutron I, Hoffmann TC, Mulrow CD, et al. The PRISMA 2020 statement: an updated guideline for reporting systematic reviews. BMJ. 2021;372:n71. doi: 10.1136/bmj.n71 33782057 PMC8005924

[34] McGowan J, Sampson M, Salzwedel DM, Cogo E, Foerster V, Lefebvre C. PRESS Peer Review of Electronic Search Strategies: 2015 Guideline Statement. J Clin Epidemiol. 2016;75:40–6. 27005575 10.1016/j.jclinepi.2016.01.021

[35] Maher CG, Sherrington C, Herbert RD, Moseley AM, Elkins M. Reliability of the PEDro scale for rating quality of randomized controlled trials. Phys Ther. 2003;83(8):713–21. 12882612

[36] MacPhail A, Kirk D, Griffin L. Throwing and Catching as Relational Skills in Game Play: Situated Learning in a Modified Game Unit. Journal of Teaching in Physical Education. 2008;27(1):100–15.

[37] Oh H-J, Bullard S, Hovatter R. Speedminton: Using the Tactical Games Model in Secondary Physical Education. Strategies. 2011;25(1):26–30.

[38] Thomas G, Morgan K, Mesquita I. Examining the implementation of a Teaching Games for Understanding approach in junior rugby using a reflective practice design. Sports Coaching Review. 2013;2(1):49–60.

[39] Smith L, Harvey S, Savory L, Fairclough S, Kozub S, Kerr C. Physical activity levels and motivational responses of boys and girls. European Physical Education Review. 2014;21(1):93–113.

[40] Harvey S, Pill S. Comparisons of Academic Researchers’ and Physical Education Teachers’ Perspectives on the Utilization of the Tactical Games Model. Journal of Teaching in Physical Education. 2016;35(4):313–23.

[41] Harvey S, Smith ML, Song Y, Robertson D, Brown R, Smith LR. Gender and School-Level Differences in Students’ Moderate and Vigorous Physical Activity Levels When Taught Basketball Through the Tactical Games Model. Journal of Teaching in Physical Education. 2016;35(4):349–57.

[42] Harvey S, Atkinson O. One youth soccer coach’s maiden implementation of the Tactical Games Model. Ágora para la Educación Física y el Deporte. 2017;19(2–3):135–57.

[43] Harvey S, García-López LM. Objectively measured physical activity of different lesson contexts. Journal of Physical Education and Sport. 2017;17(02):833–8.

[44] Williams SM, Hannon JC. Physical Activity Levels in Coed and Same-Sex Physical Education Using the Tactical Games Model. The Physical Educator. 2018;75(3):525–45.

[45] Alagül Ö, Gürsel F. Teacher who (cannot) Change: Experimental Processes of Physical Education Teachers by means of Pedagogical Innovations at the time of Their Professional Development. Ted EĞİtİm Ve Bİlİm. 2019;44(197):401–20.

[46] Garcia-Ceberino JM, Feu S, Ibanez SJ. Comparative Study of Two Intervention Programmes for Teaching Soccer to School-Age Students. Sports (Basel). 2019;7(3). doi: 10.3390/sports7030074 30917526 PMC6473480

[47] Gouveia ÉR, Gouveia BR, Marques A, Kliegel M, Rodrigues AJ, Prudente J, et al. The effectiveness of a tactical games approach in the teaching of invasion games. Journal of Physical Education and Sport. 2019;2019(3):962–70.

[48] Güneş B, Yılmaz E. The Effect of Tactical Games Approach in Basketball Teaching on Cognitive, Affective and Psychomotor Achievement Levels of High School Students. Ted EĞİtİm Ve Bİlİm. 2019;44(200):313–31.

[49] Rodríguez-Negro J, Falese L, Yanci J. Effects of different balance interventions for primary school students. The Journal of Educational Research. 2019;112(6):656–62.

[50] Coppola R, Pignato S, Sgrò F, Lipoma M. Effects of Two Different Physical Education Teaching Approaches on the Levels of Enjoyment in the Italian Primary School Students. Journal of Human Sport and Exercise. 2020;15:S1251–S61.

[51] García-Ceberino JM, Antúnez A, Feu S, Ibáñez SJ. Quantification of Internal and External Load in School Football According to Gender and Teaching Methodology. International Journal of Environmental Research and Public Health. 2020;17(1). 10.3390/ijerph17010344 PMC6981553 31947877

[52] García-Ceberino JM, Gamero MG, Feu S, Ibáñez SJ. Experience as a Determinant of Declarative and Procedural Knowledge in School Football. International Journal of Environmental Research and Public Health. 2020;17(3). doi: 10.3390/ijerph17031063 32046167 PMC7037024

[53] García-Ceberino JM, Gamero MG, Feu S, Ibáñez SJ. Differences in Technical and Tactical Learning of Football According to the Teaching Methodology: A Study in an Educational Context. Sustainability. 2020;12(16).

[54] Gonzalez-Espinosa S, Antunez A, Feu S, Ibanez SJ. Monitoring the External and Internal Load Under 2 Teaching Methodologies. J Strength Cond Res. 2020;34(10):2920–8. doi: 10.1519/JSC.0000000000002799 30216248

[55] González-Espinosa S, García-Rubio J, Feu S, Ibáñez SJ. Carga Externa SegÚn La SituaciÓn De Juego Y MetodologÍa En Baloncesto Escolar. Revista Internacional de Medicina y Ciencias de la Actividad Física y del Deporte. 2020;20(79):395–417.

[56] Rodríguez-Negro J, Yanci J. Which instructional models influence more on perceived exertion, affective valence, physical activity level, and class time in physical education? Educational Psychology. 2019;40(5):608–21.

[57] Sgrò F, Barca M, Schembri R, Lipoma M. Assessing the effect of different teaching strategies on students’ affective learning outcomes during volleyball lessons. Journal of Physical Education and Sport. 2020;2020(3):2136–42.

[58] SgrÒ F, Coppola R, Tortella P, Lipoma M. Tactical Games Model as curriculum approach at elementary school: Effects on in-game volleyball technical improvements. Journal of Human Sport and Exercise. 2020;15(4):S1178–S86.

[59] Gamero MG, Garcia-Ceberino JM, Ibanez SJ, Feu S. Influence of the Pedagogical Model and Experience on the Internal and External Task Load in School Basketball. Int J Environ Res Public Health. 2021;18(22). doi: 10.3390/ijerph182211854 34831609 PMC8623569

[60] Gamero MG, García-Ceberino JM, Ibáñez SJ, Feu S. Analysis of Declarative and Procedural Knowledge According to Teaching Method and Experience in School Basketball. Sustainability. 2021;13(11).

[61] Gonzalez-Espinosa S, Garcia-Rubio J, Feu S, Ibanez SJ. Learning Basketball Using Direct Instruction and Tactical Game Approach Methodologies. Children (Basel). 2021;8(5). doi: 10.3390/children8050342 33926068 PMC8145114

[62] Schembri R, Coppola R, Tortella P, Sgrò F. Improving enjoyment during physical education lesson in primary school students. Journal of Human Sport and Exercise. 2021;16:S735–S42.

[63] Sgrò F, Coppola R, Schembri R, Lipoma M. The effects of a tactical games model unit on students’ volleyball performances in elementary school. European Physical Education Review. 2021;27(4):1000–13.

[64] Feu S, Garcia-Rubio J, Ibanez SJ, Antunez A. External load of the tasks planned by teachers for learning handball. PLoS One. 2022;17(4):e0265745. doi: 10.1371/journal.pone.0265745 35381045 PMC8982867

[65] Garcia-Ceberino JM, Feu S, Gamero MG, Ibanez SJ. Determinant Factors of Achievement Motivation in School Physical Education. Children (Basel). 2022;9(9). doi: 10.3390/children9091366 36138675 PMC9497943

[66] Setiawan E, Juliantine T. Effect of Tactical Game Models on Formation of Basic Techniques in Handball Players: Mixed Method. Physical Education Theory and Methodology. 2022;22(3):373–8.

[67] Rodríguez-Negro J, Yanci J. Effects of two different physical education instructional models on creativity, attention and impulse control among primary school students. Educational Psychology. 2021;42(6):787–99.

[68] Hartati H, Ursa M, Hardiyono B, Jaya RW. Improving creativy and learning motivation in basketball through tactical approach. Jurnal Cakrawala Pendidikan. 2022;41(2):521–30.

[69] Sucipto S, Hambali B, Gumilar A, Nur L. Enjoyment in physical education learning: The effect of learning approach and gender. Jurnal Cakrawala Pendidikan. 2023;42(3):719–32.

[70] Gotschall T. EndNote 20 desktop version. J Med Libr Assoc. 2021;109(3):520–2. doi: 10.5195/jmla.2021.1260 34629985 PMC8485940

[71] Koekoek J, Knoppers A. The role of perceptions of friendships and peers in learning skills in physical education. Physical Education and Sport Pedagogy. 2015;20(3):231–49.

[72] Silva R, Farias C, Ramos A, Mesquita I. Implementation of Game-Centered Approaches in Physical Education: A Systematic Review. Journal of Physical Education and Sport. 2021;21(6):3246–59.

[73] Mitchell SA, Oslin JL, Griffin LL. Teaching Sport Concepts and Skills: A Tactical Games Approach for Ages 7 to 18: Human Kinetics; 2013.

[74] Barba-Martín RA, Bores-García D, Hortigüela-Alcalá D, González-Calvo G. The application of the teaching games for understanding in physical education. Systematic review of the last six years. International journal of environmental research and public health. 2020;17(9):3330. doi: 10.3390/ijerph17093330 32403272 PMC7246645

[75] Yilmaz K. Comparison of quantitative and qualitative research traditions: Epistemological, theoretical, and methodological differences. European journal of education. 2013;48(2):311–25.

[76] Stolz S, Pill S. Teaching games and sport for understanding: Exploring and reconsidering its relevance in physical education. European Physical Education Review. 2014;20(1):36–71.

[77] Wang C, Omar Dev RD, Soh KG, Mohd Nasirudddin NJ, Yuan Y, Ji X. Blended learning in physical education: A systematic review. Frontiers in public health. 2023;11:1073423. doi: 10.3389/fpubh.2023.1073423 36969628 PMC10034186

[78] Fraser-Thomas JL, Côté J, Deakin J. Youth sport programs: An avenue to foster positive youth development. Physical education & sport pedagogy. 2005;10(1):19–40.

[79] Bailey R, Armour K, Kirk D, Jess M, Pickup I, Sandford R, et al. The educational benefits claimed for physical education and school sport: an academic review. Research papers in education. 2009;24(1):1–27.

[80] Estevan I, Bardid F, Utesch T, Menescardi C, Barnett LM, Castillo I. Examining early adolescents’ motivation for physical education: Associations with actual and perceived motor competence. Physical Education and Sport Pedagogy. 2021;26(4):359–74.

[81] Morales-Belando MT, Calderón A, Arias-Estero JL. Improvement in game performance and adherence after an aligned TGfU floorball unit in physical education. Physical Education and Sport Pedagogy. 2018;23(6):657–71.

[82] Goncalves A, Deshayes M, Gisclard B, Philippe AG, Bernal C, Krawczyk S, et al. Exploring the Health Effectiveness of a Physical Activity Program Co-Constructed with Students after the COVID-19 Pandemic. Nutrients. 2023;15(13):2913. doi: 10.3390/nu15132913 37447238 PMC10346168

[83] Ramos A, Coutinho P, Leitao JC, Cortinhas A, Davids K, Mesquita I. The constraint-led approach to enhancing team synergies in sport-What do we currently know and how can we move forward? A systematic review and meta-analyses. Psychology of Sport and Exercise. 2020;50:101754.

[84] Vernadakis N, Papastergiou M, Zetou E, Antoniou P. The impact of an exergame-based intervention on children’s fundamental motor skills. Computers & Education. 2015;83:90–102.

[85] Griban G, Skoruy O, Pantielieiev K, Brytan Y, Tymchyk M, Kharchenko N, et al. Influence of physical education classes on the level of health and fitness competencies of students. International Journal of Applied Exercise Physiology. 2020(9 (12)):107–18.

[86] Ayling P. Learning through playing in higher education: Promoting play as a skill for social work students. Social Work Education. 2012;31(6):764–77.

[87] Guo Y. The Influence of Academic Autonomous Motivation on Learning Engagement and Life Satisfaction in Adolescents: The Mediating Role of Basic Psychological Needs Satisfaction. Journal of Education and Learning. 2018;7(4):254–61.

